# Recruitment of nursing homes and residents in a cluster-randomised controlled trial to improve oral health (MundZaRR): a structured analysis of trials and tribulations

**DOI:** 10.1186/s13063-026-09607-5

**Published:** 2026-03-13

**Authors:** Lisa-Maria Wobst, Jens Abraham, Sarah Habig, Saskia Gabelmann, Georg Gaßmann, Falk Schwendicke, Peter Schlattmann, Helmut Orawa, Katrin Hertrampf, Gabriele Meyer

**Affiliations:** 1 Institute of Health, Midwifery and Nursing Science, Medical Faculty of Martin Luther University Halle-Wittenberg, University Medicine Halle, Halle (Saale), Germany; 2 Clinic of Oral and Maxillofacial Surgery, University Hospital Schleswig-Holstein, Kiel, Germany; 3 praxisHochschule, University of Applied Sciences, Cologne, Germany; 4 Clinic for Conservative Dentistry and Periodontology, LMU University Hospital, LMU Munich, Munich, Germany; 5 Institute for Medical Statistics, Computer and Data Sciences, University Hospital Jena, Jena, Germany

**Keywords:** Recruitment, Cluster-randomised controlled trials, Oral health, Nursing homes, Residents

## Abstract

**Background:**

The cluster-randomised controlled MundZaRR trial aimed to improve the oral health of nursing home (NH) residents through a complex intervention programme. Recruitment in NH settings is notably challenging. Since there is only a limited number of reports from comparable studies on recruitment, we aimed to analyse the challenges and barriers related to the recruitment process of MundZaRR.

**Methods:**

NHs in Southwestern Germany were recruited by the study team, and residents via NH contact persons, e.g. NH managers. We aimed for 618 recruited residents from 18 NHs. Due to two COVID-19 pandemic-related interruptions, recruitment occurred in three phases from September 2019 until June 2022. The recruitment strategies had to be adapted during the course of the pandemic (e.g. from face-to-face to email/telephone contact). The recruitment process was continuously documented and analysed descriptively.

**Results:**

A total of 111 NHs were deemed eligible, and finally 18 NHs agreed to participate (participation rate: 16%). Overall, 358 individual consents were obtained, *n* = 89 (25%) from residents and *n* = 269 (75%) from their representatives, resulting in an average participation rate (corresponding to the NHs’ total number of beds) of 27% (min = 10%, max = 85%). Major challenges were the withdrawal of eight clusters (with *n* = 87 residents) from the first recruitment phase and the high number of residents who deceased during the recruitment process (*n* = 75). The COVID-19 pandemic, associated with time constraints and staff shortages at the NHs, was another significant barrier. At the individual level, obtaining informed consent from residents’ representatives was particularly challenging.

**Conclusion:**

Despite using various strategies and intensive efforts, we achieved the planned number of clusters, but failed to reach the targeted sample size of residents. The pandemic-related measures and negative effects as well as the process of obtaining consent from residents’ representatives were major challenges. Personal contact with NHs and flexible approaches for reaching residents’ representatives seem to be important strategies. However, future cluster-randomised studies with comparable vulnerable populations should assume a higher number of clusters and carefully pilot recruitment strategies in preceding feasibility studies.

**Trial registration:**

ClinicalTrials.gov NCT04140929. Registered on 24 October 2019. https://clinicaltrials.gov/study/NCT04140929.

## Background

In Germany, approximately 800,000 residents live in nursing homes (NHs) [1]. Residents are frequently affected by complex physical and cognitive impairments leading to dependency in the activities of daily living. Oral health complaints are particularly prevalent among NH residents, with many requiring assistance with this aspect of care [2]. The Fifth German Oral Health Study has identified that NH residents requiring care exhibit poorer dental and oral health in comparison to the related age group (75 to 100 years) without care dependency [3]. Dental caries, periodontal disease, and poorly fitting dentures are common problems faced by many NH residents [4, 5]. Left untreated, these conditions can reduce oral health-related quality of life and increase the risk of comorbidities such as malnutrition or pneumonia [5, 6]. As a result, the wide implementation of interventions to improve oral health has been suggested [2, 5, 6]. In 2010, the German Federal Dentists Association and the National Association of Statutory Health Insurance Dentists introduced the nationwide initiative *“Oral health despite handicap and high age”*, which enabled contractual cooperation agreements between dentists and NHs to facilitate structured dental care for residents. Since 2014, NHs in Germany have the opportunity to conclude such cooperation agreements, where residents are entitled to regular dental examinations in the NHs. The results and corresponding recommendations are documented on a standardised form and provided to nursing staff. This approach is aimed in particular at residents who are unable to visit the dentist regularly due to physical or cognitive impairments. Based on this concept, we conducted a parallel-group, cluster-randomised, controlled trial (cRCT), entitled MundZaRR, which aimed at investigating a complex intervention dealing with “remotivation” and “reinstruction” of nursing staff by dental assistants [7]. In both study groups, all participating NH residents were assessed by dentists, forming the basis for individual oral hygiene and care recommendations. The dentists used a standardised form, developed by the German Federal Dental Chamber, to instruct and motivate nursing staff on individual oral care measures. In the intervention group, dental assistants reinstructed and remotivated the nursing staff according to the initial recommendations of the dentist. Reinstruction comprised the support of nursing staff regarding the implementation of recommended oral hygiene measures, e.g. through guidance or advice on improving oral hygiene practices, as well as assistance in acquiring the necessary oral hygiene materials. Remotivation included conversations with nurses to increase the awareness for the topic of oral hygiene or seeking tailored strategies for individual residents. The study enrolled NH residents with moderate to high care dependency (care level three to five assessed by the medical service of the German social care insurance, including e.g. mobility, cognitive and communicative abilities, organising everyday life and social contact). Respite care was an exclusion criterion. Exclusion criteria at the cluster level were existing dental cooperation agreements (according the German social insurance §119b SGB V).

The international literature indicates several challenges in conducting studies within the NH setting, whereby recruitment has been highlighted as one of the greatest, both at the cluster and resident level [8]. A lack of experience and unfamiliarity with research, as well as a lack of interest or negative attitudes towards research, are just some of the reasons why NHs frequently decline to participate [8]. Poor response from NHs, difficulties in maintaining their interest, and low priority given to research, which turned out to make the recruitment process time-consuming and costly [9–11] have been described earlier. At the individual level, residents tend to decline study participation because they do not perceive personal benefits or fear health risks. Often residents do not trust the researchers or do not want to be disturbed in their daily routines [8, 12–14]. Since the majority of residents suffer from cognitive impairment, informed consent often has to be obtained from legal guardians, which makes the procedure more complex and time-consuming [13–16]. Due to these cumbersome conditions, the calculated sample size is often not reached, which negatively affects the trustworthiness of the study results [17]. Former RCTs investigating comparable interventions that aimed to improve oral hygiene in NH residents reported only limitedly on the recruitment process [18–21]. Therefore, we conducted a structured evaluation of the recruitment process as part of the comprehensive process evaluation of the main MundZaRR trial (will be published elsewhere). Our primary objective was to systematically describe the recruitment strategies and recruitment outcomes for NHs and residents. In addition, we explored the challenges and barriers related to the recruitment process, particularly with regard to the COVID-19 pandemic.

## Materials and methods

### Methodology of the recruitment process

The study protocol of the MundZaRR trial has been published elsewhere [7]. The sample size calculation revealed a number of 618 residents from 18 NHs. This was based on an assumed effect size from the feasibility study and adjusted for cluster randomisation (average of 34 residents per NH, intracluster correlation = 0.05). Variability in the number of residents per cluster was anticipated, and a dropout rate of about 30% due to mortality or severe morbidity was incorporated into the calculation. The recruitment process was organised in two steps: First, NHs in Southwestern Germany were invited and, subsequently, residents were recruited through contact persons of the NHs, e.g. NH managers, directors of nursing or ward managers. The randomisation of the 18 participating NHs was carried out after the last individual participant was included. Nine NHs with 178 residents were assigned to the intervention group, and nine NHs with 180 residents to the control group.

#### Recruitment of nursing homes

The recruitment of NHs for this study was carried out in three phases. The COVID-19 pandemic led to an interruption in the first phase and forced an adaptation of the recruitment strategy for the second and third phases. 

*First phase*: According to the initial recruitment strategy, only the NHs of one cooperation partner (affiliated to the church) were intended to enrol. Recruitment started in September 2019 with two kick-off events to introduce the project to the cooperation partner's NH managers. Fifteen of 28 eligible NHs had concluded a cooperation agreement during the application phase of the project and thus met the exclusion criteria. The recruitment strategy was therefore extended to other denominational and non-denominational NHs. Various techniques were applied to identify additional NHs, including searching for eligible NHs in two online databases and contacting major German welfare organisations. These welfare organisations, which are often owners of NHs in Germany, were contacted in order to gain access to a larger number of NHs. All managers of potentially relevant NHs were contacted by telephone or email, and on-site visits were arranged.

Due to the onset of the pandemic in early March 2020, the study team decided to discontinue recruitment. In April 2020, visits to NHs were even prohibited by the government.

*Second phase*: In October 2020, a second recruitment phase was initiated. Since access to NHs was still prohibited, a digital recruitment approach was developed. After an initial telephone contact with NH managers, an online meeting was offered to present the project via a video conferencing platform. However, recruitment had to pause again after January 2021 due to another pandemic-related lockdown. Continuous contact was maintained with the NHs that had already consented to participate in the previous recruitment phase. Recruitment was postponed until August 2021 for the other interested NHs that had not yet confirmed their participation for pandemic-related reasons.

*Third phase*: The third recruitment phase started in August 2021. At this time, postponed NHs from the first two recruitment phases were approached to reconfirm their willingness to participate. The managers of these NHs received an email with information about the resumption of the project and written consent forms. Study information was sent once more if needed. Moreover, new NHs were recruited. For reasons of feasibility, new potentially eligible NHs located closer to the catchment areas of the dentists already enrolled in the study were considered. Initial contact with NH managers was mainly carried out by telephone. Eligible NHs received information material via email. If necessary, a further appointment was scheduled for a detailed discussion and project presentation, either by telephone or online. Additionally, NH managers were asked to suggest further eligible NHs via snowball sampling technique. All NHs expressing interest were provided with a written consent form. Once a NH had consented, project information, consent forms, and reply-paid envelopes for resident recruitment were sent to the NHs by post. Financial compensation or other incentives were not provided.

#### Recruitment of residents

In each NH, recruitment of residents started after written consent had been obtained from the NH management. For data protection reasons, the initial information and recruitment of residents were carried out by the contact person (NH manager, director of nursing, or ward manager) of each NH. The responsible contact person was asked to identify eligible residents based on the inclusion criteria and to assess their ability to give consent. Consequently, residents were informed about the project and provided with written project information and consent forms. The informed consent included the collection of data including dental examinations as well as the potential treatment according to the cluster allocation. For residents who were unable to consent due to cognitive impairments, the written project information and consent forms were forwarded to their legal guardians. The contact persons of the NHs were responsible for contacting the legal guardians and distributing the written information and consent documents. Various approaches were applied, such as the delivery of the information and consent forms by postal mail, handing them over in person, or conducting an information event on-site. Reminder documents were provided to NHs in case of non-responders. The study team was approachable by telephone to answer questions or other concerns. To maintain contact with the NHs and to support resident recruitment if necessary, the NHs were contacted by phone at regular intervals.

### Data collection and analysis

The recruitment of NHs and residents was carefully documented as a part of the process evaluation of the study using a structured protocol. The documentation of the individual phases of the NH recruitment process included approvals and refusals from NHs, number and methods of contacts, and additional field notes regarding possible facilitators or barriers. Reasons for non-participation were only documented if NHs were willing to provide reasons voluntarily.

Since the contact persons on site were in charge of residents’ recruitment, only the number of informed consents from residents or their legal guardians per NH was available, but not the total number of residents approached.

Quantitative data was analysed descriptively and presented as frequencies or mean values. Qualitative data from free text responses was categorised and presented as frequencies or described narratively.

## Results

### Recruitment of nursing homes

**Fig. 1:**
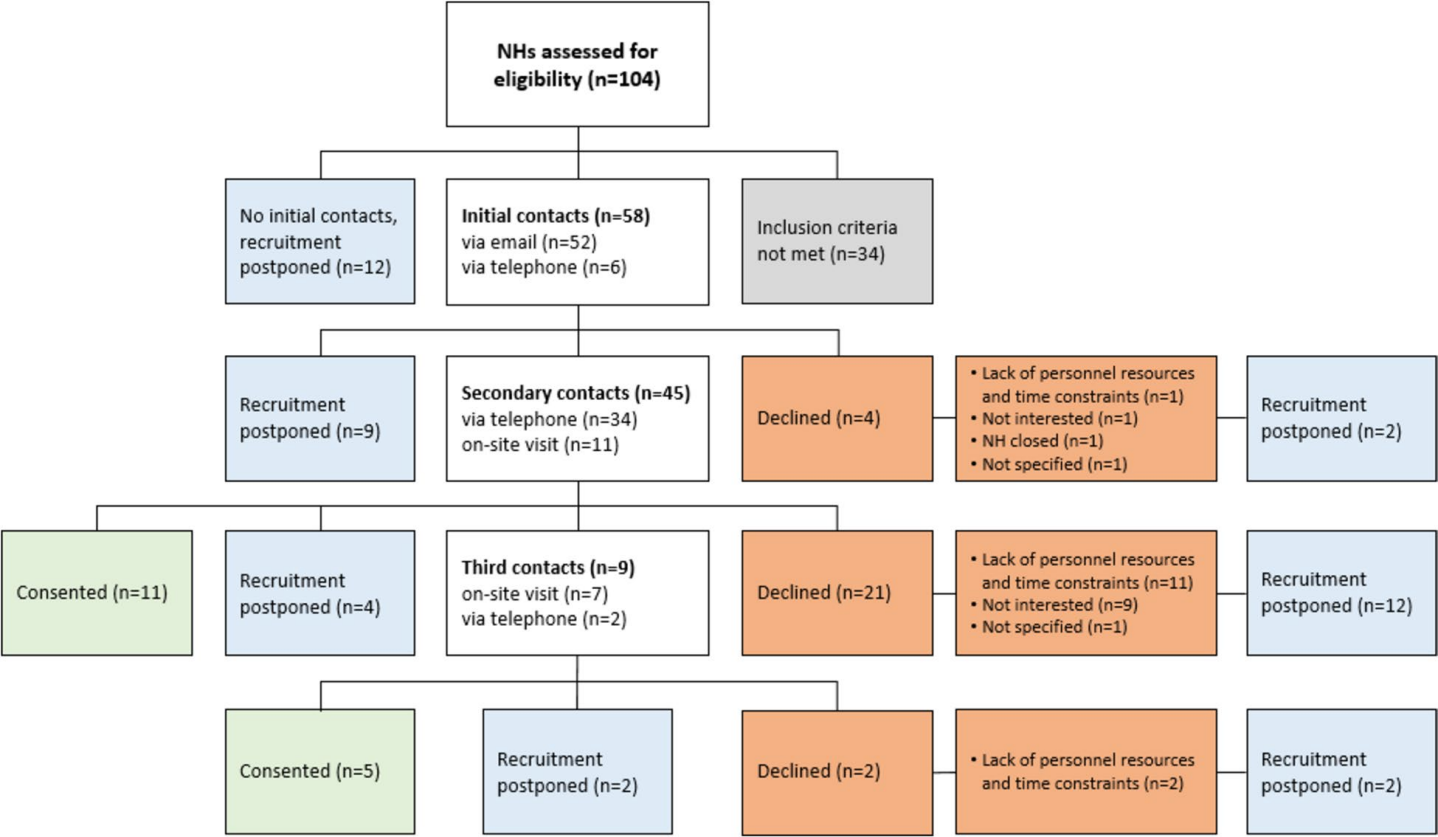
Flow chart of nursing home recruitment in the first phase (September 2019 until March 2020). Different box colours indicate different recruitment decisions (blue: recruitment postponed, orange: declined participation and reasons for non-participation, green: consented participation, grey: inclusion criteria not met)

In the first phase (September 2019 until March 2020), 104 NHs were assessed for eligibility, of which 34 did not meet the inclusion criteria. Initial contacts were made with 58 NHs, mainly via email (*n* = 52). As part of the secondary contacts (*n* = 45), eleven NHs were visited by a project member on-site and all visits resulted in a consent from the NHs. Thirty-four NHs were contacted via telephone, most of them declined participation (*n* = 21). Third contacts were required for nine NHs (*n* = 7 on-site visits, *n* = 2 telephone), which led to another five consents (*n* = 4 following on-site visits). Recruitment was postponed for 27 NHs, mainly because initial contacts could not be established or because the NHs requested that the enquiry be repeated at a later date. Additionally, sixteen NHs that declined participation due to general pandemic-related challenges were reapproached during NH recruitment in phase 3. As previously described, recruitment had to be paused in March 2020 due to the onset of the pandemic and associated visiting bans in NHs. Figure 1 shows the flow of NH recruitment in the first phase, as well as detailed reasons for non-participation.

**Fig. 2:**
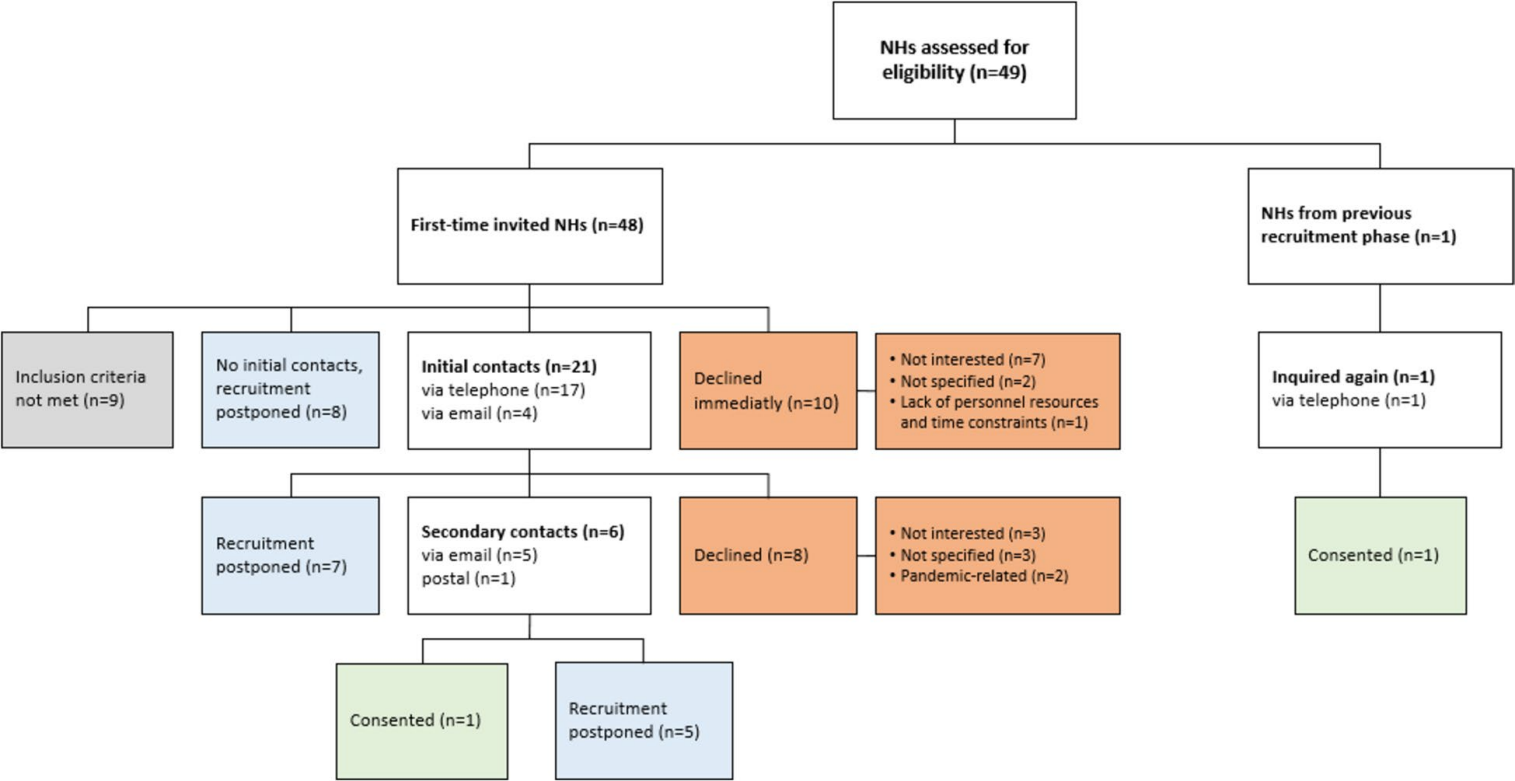
Flow chart of nursing home recruitment in the second phase (October 2020 until January 2021). Different box colours indicate different recruitment decisions (blue: recruitment postponed, orange: declined participation and reasons for non-participation, green: consented participation, grey: inclusion criteria not met)

As described in the methods section, a digital recruitment approach was offered in the second phase (October 2020 until January 2021). However, none of the NHs contacted opted for an online study presentation. Forty-eight new NHs were assessed for eligibility; nine of them were found to be ineligible. Ten NH managers declined immediately upon the first contact attempt without requesting further information about the study. Initial contacts were made with 21 NHs (*n* = 17 telephone, *n* = 4 email), and six NHs were contacted twice (*n* = 5 email, *n* = 1 by post), resulting in one consent. Another consent was obtained from a NH that had already been contacted in the first phase and was now approached once more. It soon became evident that many NHs were still affected by the pandemic and were therefore unable to take part in the study. As a result, recruitment was halted again in January 2021 after consultation with the project sponsor, and recruitment for 20 NHs was postponed. The recruitment process of the second phase is displayed in Fig. 2.

In the third phase (August 2021 until June 2022), two main NH recruitment approaches were employed. The first approach involved assessing the eligibility of 79 NHs from previous recruitment phases. Among these, 61 were postponed NHs, of which 25 were deemed eligible and were contacted via telephone. Only two agreed to participate. The remaining 18 NHs, which had already agreed to participate during the first and second phases, were asked again to reconfirm their willingness to participate. Nine of them consented again, while the other nine NHs withdrew from participation.

**Fig. 3:**
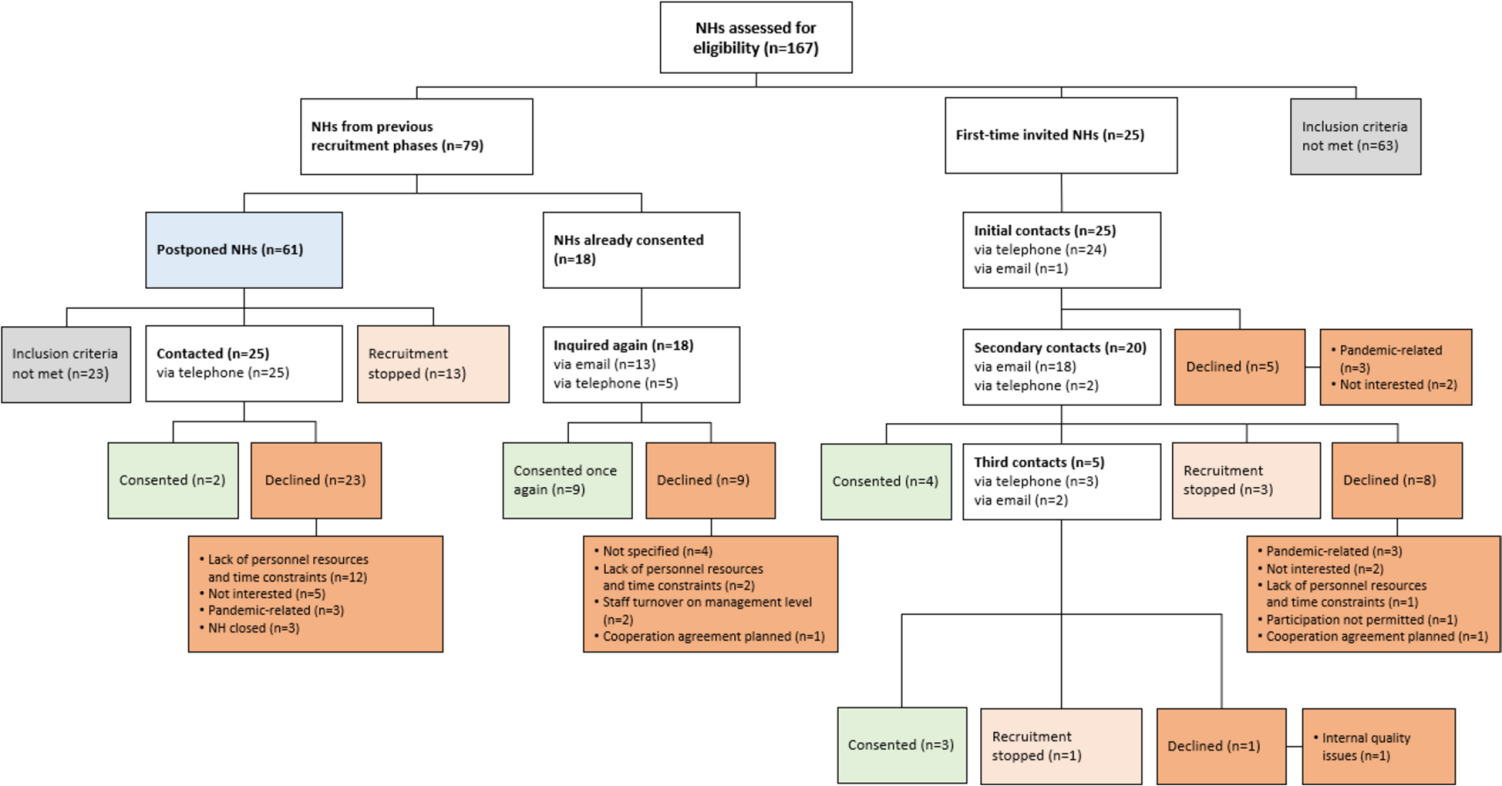
Flow chart of nursing home recruitment in the third phase (August 2021 until June 2022). Different box colours indicate different recruitment decisions (blue: recruitment of postponed nursing homes, orange: declined participation and reasons for non-participation, pale orange: recruitment stopped, green: consented participation, grey: inclusion criteria not met)

The other approach focused on recruiting new NHs. Therefore, 25 NHs were contacted for the first time (*n* = 24 telephone, *n* = 1 email). After a secondary contact with 20 NHs (*n* = 18 email, *n* = 2 telephone), four agreed to participate. Five NHs were approached for a third time (*n* = 3 telephone, *n* = 2 email), resulting in another three consents. For a total of 17 NHs, recruitment was stopped, primarily due to their unavailability or insufficient time before the start of the cRCT. The respective flow of NH recruitment in the third phase and reasons for non-participation are presented in Fig. 3.

By the end of the third phase, a total of 139 NHs had been contacted, 111 were deemed eligible, and finally 18 NHs agreed to participate. The participation rate at the cluster level was calculated from the total number of potentially eligible and finally recruited NHs, resulting in a recruitment rate of approximately 16%.

Fifty-eight NHs provided reasons for non-participation, with some giving multiple responses. The pandemic was a frequently mentioned reason for non-participation, as it posed major challenges for NHs and was often accompanied by time constraints and staff shortages. Lack of interest was also repeatedly mentioned. Other reasons were, e.g., the involvement in other research projects, internal quality issues within NHs, or the planned conclusion of the cooperation agreement.

### Recruitment of residents

During the first phase, 13 out of 16 enrolled NHs had already started intensive recruitment of residents. However, three NHs, which had agreed to participate in the study towards the end of the first phase, returned only a small number of consent forms from residents or their legal guardians. A total of 249 individual consent forms were received during this phase.

Due to the pandemic, recruitment of residents could not be continued during the second phase.

With the start of the third phase, resident recruitment proceeded. As written above, all eighteen NHs, which had agreed to participate in previous recruitment phases, were contacted again. Consequently, nine NHs (*n* = 8 NHs of the first phase and *n* = 1 NH of the second phase) withdrew their participation, which caused a loss of 87 residents. Additionally, 75 residents were deceased, two had moved to another place, and one withdrew consent. The remaining 84 residents or their legal guardians were contacted again regarding participation in the study, with 42 consenting once again. Furthermore, 115 new participants were recruited from the remaining eight NHs of the first phase. One NH, which had already been recruited in the second phase, began recruiting residents for the first time in the third phase. This NH, as well as nine newly recruited NHs of the third phase, provided a further 201 consents from residents or their legal guardians.

At the end of resident recruitment in the third phase, a total of 358 individual informed consents had been obtained from 18 NHs. Obtaining the consent from legal guardians proved to be challenging. Only 89 consents (25%) were given by the residents themselves, while 269 (75%) were provided by legal guardians. Although the sample size was considerably lower than calculated, the recruitment period was not further extended, particularly due to the high number of deceased residents. Between the end of resident recruitment and baseline data collection, there was a rate of 24% (*n* = 87) loss of recruited residents. The main reasons were death (*n* = 45), subsequent exclusion due to ineligibility (*n* = 14) or withdrawal (*n *= 8). Eventually, the final sample size at baseline data collection was 271 residents.

The analysis of the participation rates of residents for the first phase and the third phase revealed comparable results. As no data was available from the NHs on the number of potentially eligible residents who met the inclusion criteria, the participation rate at resident level was calculated from the individual total number of NH beds and the total number of residents who consented. In the first phase, 16 NHs were enrolled until recruitment was halted. As mentioned previously, three of these NHs only yielded a small number of consents up to that time. The mean recruitment period per NH was 5 months (min = 3, max = 7). During this phase, the participation rate was 19%. As no residents were recruited in the second phase, no corresponding participation rate could be calculated. In the third phase, eight NHs of the first phase continued to recruit residents, whereby some losses of already recruited residents had been recorded in the meantime. The recruitment period for these NHs was extended to an average of 14 months, resulting in a participation rate of 29%. Compared to the 16 NHs in the first phase, the ten newly recruited NHs of the second and the third phase also had an average recruitment period of five months (min = 3, max = 9), yielding a participation rate of 20%. 

At the end of the recruitment in the third phase, the overall participation rate of residents from the 18 recruited NHs was 27%, with large differences between the individual NHs, ranging from a minimum of 10% (*n* = 15/151) to a maximum of 85% (*n* = 23/27). The individual participation rates for all NHs are displayed in Table 1.

**Table 1:** Resident participation rates (from highest to lowest participation rate)

Nursing home	Nursing home beds (*n*)	Residents recruited (*n*)	Participation rate (%)
1	27	23	85
2	65	31	48
3	24	9	38
4	59	22	37
5	90	25	28
6	113	31	27
7	34	9	27
8	61	14	23
9	87	19	22
10	117	26	22
11	131	28	21
12	40	8	20
13	98	19	19
14	88	17	19
15	100	17	17
16	190	32	17
17	103	13	13
18	151	15	10

### Further challenges and barriers of the recruitment process

By analysing the field notes and open-ended information from the recruitment protocol, several challenges and barriers were identified at the cluster and individual levels.

As reported above, the COVID-19 pandemic was a significant barrier to the entire recruitment process. Many of the contacted NHs experienced time constraints and a lack of personnel resources, resulting in limited prioritisation of study participation. Consequently, recruitment had to be postponed several times for certain NHs. Additionally, as a consequence of the extended duration of the recruitment process, some of the already recruited NHs from the first phase were no longer able or willing to participate in the study at the time of the re-inquiry in the third phase. For those NHs that consented once again, it was necessary to reobtain a written consent from NHs and residents.

Contacting the NHs was often difficult, as the NH managers were frequently unavailable, requiring multiple contact attempts. Other NHs did not respond to any contact attempts or could no longer be reached during recruitment. In addition, study documents occasionally had to be sent repeatedly because NH managers explained that they had not received them or had not taken notice. Another barrier was staff turnover at the management level, which led to delays in the decision-making process or resulted in withdrawal of consents due to the lack of interest of the new contact person.

At the individual level, the large number of residents with cognitive impairments in the study population required the agreement and consent from legal guardians. This proved to be very time-consuming and resource-intensive and relied heavily on the cooperation and commitment of the NH contact persons. As outlined in the methods section, the recruitment of residents was carried out by the designated contact persons within the NHs. In all recruited NHs, the first consent forms from residents were received by the study team no later than the month following NH consent. However, the individual recruitment efforts of the NHs varied throughout the phase of resident recruitment. Therefore, poor NH engagement posed another significant barrier, as the written consent forms from the residents were sometimes returned delayed or not at all. In four NHs, this ultimately led to an exclusion from the study. Additionally, withdrawals of clusters and the mortality of the target group necessitated the recruitment of new residents.

## Discussion

The aim of this study was to identify, describe and analyse the challenges and barriers associated with the recruitment of NHs and residents for a cluster-randomised controlled trial to improve oral health in NH residents. The recruitment process of the MundZaRR study from the inclusion of the first NH until the inclusion of the last resident lasted a total of 22 months. Due to the global pandemic, recruitment had to be interrupted twice for several months between March and October 2020, and again from February until August 2021.

During the initial recruitment phase, 16 NHs and 249 residents were recruited. Two additional NHs were recruited during the second recruitment phase. The extended periods between recruitment phases led to the loss of some previously enrolled NHs and residents, necessitating re-recruitment. By the end of recruitment in June 2022, 18 NHs had been successfully enrolled, with 358 individual informed consents obtained from residents or their legal guardians. Although the planned number of clusters was achieved, the sample size was significantly lower than calculated.

Recruiting participants for clinical studies in NH settings has been widely discussed in the international literature as a significant challenge [8, 22, 23]. However, there is a lack of clinical studies from German-speaking countries as well as studies dealing with comparable interventions that offer insights into recruitment problems or challenges.

### Recruitment of nursing homes

In the initial phase of NH recruitment, the first barrier was related to the inclusion and exclusion criteria of the study. Several NHs affiliated with our cooperation partner had already concluded dental cooperation agreements with individual dentists during the application and approval period of the study and were thus ineligible for study participation (as this was an exclusion criterion); the same problem occurred during the other two recruitment phases. Given the increasing number of NHs with such agreements in place [24], this barrier is likely to persist and increase. Thus, by the end of 2022, 6533 NHs had concluded such agreements, which accounted for approximately 38% of all NHs in Germany [25].

Furthermore, the study dentists were recruited at an early stage of the study, which was why we decided to focus on those dentists’ catchment areas for recruiting NHs, especially in phase 3. This adjustment may have reduced the number of potentially eligible NHs; nevertheless, it was necessary for the feasibility of the study.

As in many other studies, particularly in research with patients and NH residents, the most significant challenge for recruitment arose with the onset of the COVID-19 pandemic in Germany in February 2020. Two national surveys conducted among health service and health science researchers regarding the impact of the COVID-19 pandemic on their research processes revealed that many projects faced issues with recruitment and achieving the required sample sizes [26, 27]. In our study, numerous contacted NHs were severely affected by the pandemic, which was reflected in time constraints and a lack of personnel resources. As a result, some NHs had to be contacted several times and recruitment had to be postponed repeatedly. However, the final participation rate at the end of NH recruitment was 16%. This is comparable with other studies in the NH setting conducted before the pandemic, with reported participation rates ranging from 5 to 22%, but the total number of NHs contacted varied considerably [9, 14, 28, 29].

The pandemic also necessitated an adaptation of the originally planned recruitment strategy. Since the restrictions of visiting NHs were imposed in April 2020, recruitment could only be conducted via telephone, online or email. Previous research [30, 31] has recommended conducting on-site visits, as these visits can be helpful in outlining the benefits of a project and its impact on the residents and staff to the NH managers. Furthermore, any queries or concerns regarding participation in the study can be discussed directly with the study team. It is also recommended to involve as many members as possible of the leadership team and other relevant staff in these meetings [30] and to communicate clearly and openly about the time commitment and tasks involved for NH staff [10]. The development and maintenance of relationships with NHs is of central importance in this context, as it has the potential to positively influence the willingness of NHs to participate and their further retention. Studies conducted in the USA have shown that personal visits can increase participation rates at the NH level [10, 29].

Due to the prolonged recruitment period, sustaining the NHs’ willingness to participate was challenging. A similar experience was described in a study from the USA, where the period between initial interest and receipt of written consent was identified as most critical [9]. To maintain the interest of NHs, it was necessary to establish frequent contact, and respond flexibly and sensitively to the needs of the NH managers. Previous experience or familiarity of researchers with the work and routines in the NH setting also proved to be beneficial in this respect in various studies [9, 10, 13, 23].

Other studies [28, 29, 32] have also reported that contacting NH managers was often difficult, as they were either unavailable or arranged call-backs were not carried out. Furthermore, written study information and materials were not always acknowledged or did not reach the intended recipients. Consequently, numerous contact attempts were often necessary, and study materials had to be sent several times before reaching the relevant decision-makers. Occasionally, no contact was possible with NHs for weeks, which led to recruitment being discontinued. In this context, contacting NH organisations or larger corporations that own several NHs might be helpful, as they can provide access to numerous NHs and enable the recruitment of a larger number of NHs in a shorter period of time [9, 10, 28]. We were able to recruit several NHs through our initial cooperation partner. However, as the study progressed, the approach of contacting welfare organisations proved to be unsuccessful, mainly due to a lack of response from the contact persons.

A variety of monetary and non-monetary incentives were suggested in previous studies to enhance the participation and retention of NHs [13, 30, 33, 34]. None of the NHs in our study received financial compensation or other incentives for participation. This approach could possibly be considered for future studies.

Despite a lack of personnel resources and time constraints, a lack of interest was another main reason for non-participation in our study. Previous studies have already acknowledged that interest in the research topic and the perceived fit into the NH’s practices or routines are critical for NH participation [8, 35]. However, since more detailed information was missing, it is uncertain whether the lack of interest of NHs in our study was related to the topic of oral health or to clinical research in general.

### Recruitment of residents

The power calculation for the MundZaRR study determined a sample size of 618 residents, drawn from 18 NHs with approximately 1200 residents. This corresponds to an average participation rate of about 52% per NH. However, the actual participation rate at resident level in our study was 27%. Comparable cluster-randomised studies have reported participation rates ranging from 23 to 80% [14, 20, 29]. In contrast to our study, the participation rate was calculated from the total number of residents contacted and the number of consents obtained. Given the unavailability of these data, we estimated the participation rates using the total number of beds in the NHs, which probably resulted in an underestimation. We observed considerable variations in resident participation rates across NHs, with the smallest NH achieving the highest participation rate, while the largest NH showed the lowest participation rate. Nevertheless, no systematic size-related trend could be identified.

A significant barrier regarding the resident recruitment was the inability of many eligible residents to provide informed consent due to cognitive impairments, which necessitated obtaining consent from legal guardians. In our study, 75% of informed consents were obtained from legal guardians, compared to rates of 43% [28] and 64% [14] in other studies. Consistent with previous studies [14, 29, 34], the process of contacting legal guardians and obtaining their informed consent proved to be challenging and protracted. Legal guardians were frequently difficult or impossible to reach, consent forms were filled in incorrectly or illegibly, or it took a very long time for the consent documents to be returned. As a result, repeated contact with NH staff or follow-up queries were often necessary. Additionally, at the beginning of the study, some residents and legal guardians declined participation, presuming financial costs. In general, dental care for NH residents in Germany is covered by the statutory health insurance, which reimburses most preventive and necessary curative treatments. Co-payments are only required for services which go beyond standard care (e.g., aesthetic, customised and high-quality dentures). Participation in the MundZaRR study and the associated dental examinations did not involve any additional costs for the residents. Consequently, the information material was revised and explicitly stated that participation was free of charge. Studies in the USA [28, 29] have recommended personal contact with relatives and legal guardians by study staff to address mistrust and clarify any concerns and thereby increase participation rates. However, in Germany, data protection regulations pose challenges, necessitating contact through NH staff. A strategy to facilitate personal contact could be on-site information events for relatives and legal guardians [14]. Nonetheless, this approach still relies on the involvement and coordination of NH staff. Overall, NH staff play a central role as gatekeepers in the recruitment of residents. However, factors such as staff workload, individual attitudes towards the research project and perceived relevance of the study may shape how actively resident recruitment is supported. This, in turn, leads to potential bias, as it affects the intensity and selectivity of recruitment. Future studies should therefore develop strategies to adequately inform NH staff about participant selection and ensure sufficient support from study staff.

As described above, direct access to NHs and the establishment of relationships with NH managers can have a significant impact on the recruitment process. In addition to the positive effects on the participation rates of NHs, studies have also reported positive effects on resident recruitment [10, 31, 33]. By fostering trust and understanding of the study aims, these personal contacts can increase managers’ engagement and support for the trial, e.g., by integrating recruitment activities into routine workflows and providing the necessary time and staff resources. Since April 2020, however, strict visitation restrictions were imposed in German NHs due to the COVID-19 pandemic. Initially, visits were generally prohibited, but from May 2020 onwards, they were gradually allowed again for relatives, albeit under strict conditions (e.g. a limit of one visitor per resident per day, mandatory face mask and time restrictions). Access for non-relative visitors has been permitted again since August 2021, but only with proof of vaccination or a same-day negative corona test result and depending on local COVID-19 incidence rates. In February 2023, all visitation restrictions were completely lifted. Consequently, contact with NH managers in our study was mostly restricted to telephone or email. Nevertheless, comparable participation rates at resident level were observed for the first and third recruitment phases. This might be attributed to the study team’s decision, based on their experiences from the first recruitment phase, to maintain intensive contact with both previously and newly recruited NHs. Without these frequent contacts, participation rates might have been even lower. However, it remains uncertain whether the participation rate of NHs in the third phase could have been increased through additional personal contact.

Another aspect that influenced the recruitment of residents was the increased mortality within this vulnerable study target group. In Germany, there is a trend towards shorter length of stay in NHs, particularly for residents with higher care needs, often less than 2 years [36]. This reflects that individuals tend to enter NHs at a higher age and with higher care dependency, which contributes to lower survival times after admission [37]. In our study, approximately 30% of the 249 residents recruited in the first phase deceased by the time recruitment continued in September 2021. Analyses from Germany indicate excess mortality in the target group during the COVID-19 pandemic, overlapping with the timeframe of our resident recruitment [38]. However, as we did not collect systematic data on causes of death in our study, it remains uncertain whether the pandemic contributed to a higher mortality in our study population.

Taken together, our findings suggest that many of the recruitment barriers observed in the MundZaRR study, such as limited reachability of NH managers, competing priorities, and reliance on individual NH staff engagement, are rather structural and have been described in earlier research. The impact of the COVID-19 pandemic was twofold. Firstly, it exacerbated existing contextual factors, such as staff shortages and workload, as well as the willingness to participate due to the generally uncertain situation for NHs during the pandemic. Secondly, it posed additional challenges, particularly with regard to the required interruptions of the recruitment process and discontinuation of on-site visits due to visiting bans in the NHs.

### Limitations

The results of the present study must be considered in the light of some limitations. The UK Medical Research Council framework for the development and evaluation of complex interventions recommends piloting recruitment as part of a feasibility study [39]. However, in the feasibility study that preceded the MundZaRR study, study procedures such as dental examinations and the collaboration and support of DAs and NH staff were tested, but recruitment was not piloted. Consequently, key uncertainties regarding achievable recruitment rates, required time and staffing resources, and timelines for NH and participant enrolment could only be identified during the main trial. This limited opportunities for early adaptation of recruitment strategies and may have contributed to the recruitment challenges observed. While the COVID-19 pandemic posed additional challenges in our study that could not have been foreseen when piloting the recruitment process, the experiences from this study underline the importance of piloting recruitment strategies in future trials.

Due to data protection regulations, the reasons for non-participation at the NH level could only be assessed if these were given voluntarily and independently by the NHs. Therefore, these reasons may not be representative for all NHs concerned. As the recruitment of residents was carried out solely by NH staff, there was also no information available on the total number of potentially eligible or contacted residents per NH or on individual reasons for non-participation. However, although our study focused on the topic of oral health and was severely affected by the impact of the COVID-19 pandemic, several findings—such as challenges in maintaining the interest and engagement of NHs or obtaining consent from legal representatives—may be relevant and transferable to other studies in the NH setting.

## Conclusion

The MundZaRR study encountered several challenges and barriers during the recruitment process. Despite using various strategies and intensive efforts, we achieved the planned number of clusters but failed to reach the targeted sample size.

The onset of the pandemic necessitated an adaptation of our initial recruitment strategy, as direct access to NHs became unfeasible. However, direct access to NHs and the establishment of personal relationships with NH managers seem to be critical for successful recruitment, both at the cluster and individual level. In our study, increased personal contact during the third recruitment phase may have resulted in a higher sample size at resident level. Regarding these operational aspects, close collaboration with NH management through personal contact may facilitate recruitment and should therefore be considered in future studies. Additionally, appropriate incentives may further support the willingness to participate and recruitment efforts.

Considering the design of future studies involving similarly vulnerable populations, alternative consent models or planning for a higher number of clusters could help to account for potential recruitment shortfalls. Furthermore, as recruiting residents via legal guardians proved to be particularly complex and time-consuming, flexible strategies for approaching legal guardians that are tailored to the conditions of individual NHs should be used. From an ethical perspective, substantial efforts should be made to ensure the inclusion of residents with cognitive impairment. Careful piloting of the recruitment strategies is essential for assessing their effectiveness, identifying potential barriers, and making realistic assumptions about participation rates.

## Data Availability

The data used and/or analysed during the current study are available from the corresponding author on reasonable request.

## Abbreviations

cRCT: Cluster-randomised controlled trial
NH: Nursing home
